# Fibrinogen at admission is an independent predictor of mortality in severe sepsis and septic shock

**DOI:** 10.1186/cc13399

**Published:** 2014-03-17

**Authors:** M Azfar, M Khan, M Khurshid

**Affiliations:** 1 King Saud University, Riyadh, Saudi Arabia

## Introduction

Coagulation abnormalities are common in severe sepsis or septic shock [1].

## Methods

A prospective observational cohort study of 100 patients above 18 years of age diagnosed with severe sepsis or septic shock on admission. The first blood sample collected on admission was analyzed. Data were collected through a predesigned pro forma.Those with previous history of any coagulation disorders were excluded.

## Results

Univariate analysis showed significant correlation of APACHE II, platelet, PT, aPTT, fibrinogen and D-dimer with mortality in patients with severe sepsis or septic shock. Multivariate analysis showed APACHE II>20 (*P *= 0.001), fibrinogen <2 (*P *= 0.019) and D-dimer >1(*P *= 0.06) are independent predictors of mortality in severe sepsis or septic shock. See Table 1

**Table 1:** 

Variable	Died (*n *= 65)	Survived (*n *= 35)	ARR	95% CI of ARR	*P *value
Platelet <15C	33 (50.8)	10 (28.6)	1.37	0.03, 1.84	0.03
PT >16	41 (63.1)	14 (40)	1.40	1.02,1.91	0.027
aPTT >40	41 (63.1)	14 (40)	1.40	1.02,1.91	0.027
Fibrinogen <2	40 (61.5)	15 (42.9)	1.31	0.96, 1.70	0.07
D-dimer >1	64 (98.5)	31 (88.6)	3.37	0.48, 19.5	0.03
APACHE II >20	53 (81.5)	18 (51.4)	1.80	1.15, 2.84	0.002
Age >65	27 (41.5)	8 (22.9)	1.60	1.04, 2.46	0.02

## Conclusion

The fibrinogen level at admission is an independent predictor of mortality in patients with sepsis or septic shock. It also confirms correlation of other coagulation abnormalities with the outcome.
